# Supplementary material for: A de novo missense variant in MIDEAS results in increased deacetylase activity of the MiDAC HDAC complex causing a neurodevelopmental syndrome
Source: Nat Commun. 2025 Nov 25;16:10472. doi: 10.1038/s41467-025-65472-x (PMC12647621; doi:10.1038/s41467-025-65472-x)
Supplement: Supplementary file 1 — Supplementary Information [file 41467_2025_65472_MOESM1_ESM.pdf]

Supplementary Information

A de novo missense variant in MIDEAS results in increased deacetylase activity of the MiDAC HDAC complex causing a neurodevelopmental syndrome.

Louise Fairall[1*], Kristupas Sirvydis[1*], Robert E Turnbull[1*], Suzan JG Knottnerus[2*], Oksana Gonchar[1], Frederick W Muskett[1], Rebekah Jukes-Jones[1], Lonneke van Brussel[2], Ellen van de Geer[2], Koen van Gassen[2], Paul Badenhorst[3], Diana Johnson[4], Paulien A Terhal[2], Peter M van Hasselt[5], Richard H van Jaarsveld[2^], John WR Schwabe[1^]

* These authors contributed equally

^ Co-corresponding authors

1. Institute for Structural and Chemical Biology, Department of Molecular and Cell Biology, University of Leicester. LE1 7RH. UK.

2. Department of Genetics, University Medical Center Utrecht, Utrecht, The Netherlands.

3. Institute of Cancer and Genomic Sciences, University of Birmingham, Edgbaston. B15 2TT. UK.

4. Department of Clinical Genetics, Sheffield Children's NHS Foundation Trust, Sheffield, S5 7AU. UK.

5. Department of Metabolic Disease, Wilhelmina Children's Hospital, University Medical Centre Utrecht, Utrecht, the Netherlands.

Supplementary Note 1

Proband 1

We evaluated a 26-year-old male, the second child of non-consanguineous parents. Weight and height were normal at birth, 3000 gram and 48 cm respectively. He presented at the age of 4 months with a failure to thrive, craniofacial dysmorphisms, a mild motor delay and feeding difficulties. During infancy around the age of 2-3 years contractures of all joints became clear with progressive stiffening of joints and thickening of the skin. His speech development was delayed with dysarthria, nasal speech due to velopharyngeal insufficiency and phonological abnormalities. He had impaired speech intelligibility. At age 16 years he underwent pharyngoplasty. Tone audiometry was performed several times and showed at the age of 18 years a mixed (mainly perceptive) bilateral hearing loss of 40 dB on the right side and 50 dB on the left side. There was an air-bone gap for the lower frequencies of 5-10 dB on the right side and 10-20 dB on the left side. The postnatal growth was poor with height below target height (TH-SDS). Height at adult age was 140 cm (-6.18 SD with TH-SDS around -1,89 SD). A disproportionate short stature was noticed with relatively short limbs. Sitting height/length ratio at adult age was 0.57 (4.46 SD).

Craniofacial dysmorphisms included ptosis, narrow palpebral fissures, limited facial expression, a small mouth, micrognathia and attached ear lobes. From infancy he had chronic watery diarrhea (1-3 times a day). After the age of 11 years he experienced several attacks of colicky abdominal pain, initially treated conservatively. Around the age of 21 years a hemicolectomy was done because of a volvulus of the coecum. After this he underwent several laparoscopies because of severe recurrent abdominal pain with recurrent volvulus and ileus. Histological examination of a specimen from the gut derived from the surgery showed diminished staining with antibodies for alpha-smooth muscle actin, indicated as possibly fitting a form of chronic idiopathic intestinal pseudo-obstruction. Radiological skeletal survey showed mild mesomelic shortening of the forearms and a narrowing of interpeduncular distance of L5 compared to L4. An ultrasound of the abdomen showed relative small kidneys. Magnetic resonance imaging (MRI) of the brain at age 14 years showed no relevant abnormalities. Metabolic analysis (including untargeted metabolomics) was normal. Echocardiography showed a patent ductus arteriosus that closed spontaneously, cardiological evaluation was normal at the age of 6 years. Ophthalmological examination at the age of 12 years showed mild keratitis sicca and atypical gray papillae with normal visual development. Whole exome sequencing (WES) analysis in the affected individual and his parents showed a heterozygous de novo c.1961A>C (p.Tyr654Ser) variant in *MIDEAS*.

Proband 2

We identified another affected proband, a nine-year old male, with the exact same p.Tyr654Ser (c.1961A>C) variant in *MIDEAS* using the GeneMatcher platform. He had a normal birth weight. He presented with respiratory distress as a neonate and was seen as a ward referral at 7 days when dysmorphic features were noted. He required 2 days of ventilation and 7 days Continuous Positive Airway Pressure (CPAP). Social smile was on time but there were subsequent delayed milestones. Walking at 22 months old and single word speech at 6 years.

He had a short stature (-1.35 SD) for his age, although his growth was clearly less affected than in affected individual 1. He developed speech around the age of six years. He was diagnosed with autism spectrum disorder. Because of feeding difficulties he received nasogastric tube feeding. Percutaneous endoscopic gastrostomy feeding was required from the age of 20 months to age seven years. He had diarrhea from the age 3-6 years. whilst he was receiving enteral feeds. Now he generally has a normal bowel movement once a day, the diarrhoea recurrs if he is unwell. Like proband 1, he had joint contractures of his knees noticed at 10 months, non progressive at age of 8 years. Craniofacial dysmorphisms were very similar to affected proband 1 and included narrow palpebral fissures, a low hanging columella, a small mouth, and limited facial expression. Echocardiography showed a neonatal diagnosis of mild biventricular hypertrophy and a patent ductus arteriosus that closed spontaneously. MRI scan was done at 3 months and showed a structurally normal brain with a possible slight hypomyelination for his age. Metabolic analysis (including VLCFA, plasma and urine amino acids, glucose and lactate) was normal.

Whole exome sequencing (WES) analysis in the affected individual and his parents showed a heterozygous de novo c.1961A>C (p.Tyr654Ser) variant in *MIDEAS*.

a

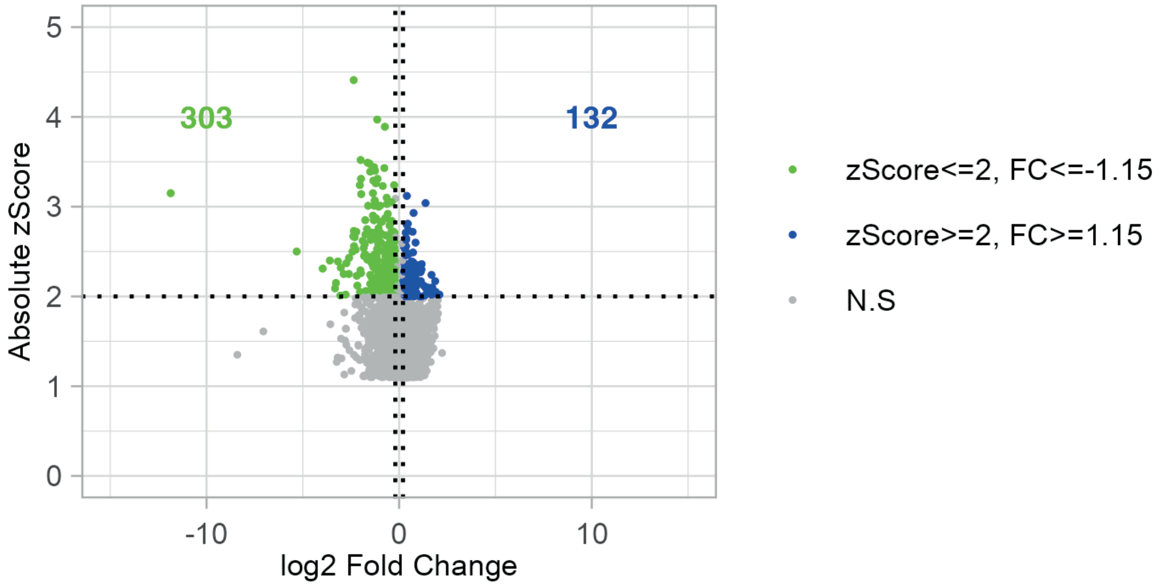

b

Gene Name	Gene Description	Uniprot ID	GO.ID	GO Name	Proband1	MEF_MIDko
HAS2	hyaluronan synthase 2	Q92819	GO:0005886	plasma membrane	1.33	-1.04
NDUFA7	NADH:ubiquinone oxidoreductase subunit A7	O95182	GO:0005739	mitochondrion	0.85	-0.15
ABCA5	ATP binding cassette subfamily A member 5	Q8WWZ7	GO:0016020	membrane	0.8	-0.40
C1orf198	chromosome 1 open reading frame 198	Q9H425	GO:0005737	cytoplasm	0.78	-0.32
CDKN2A	cyclin dependent kinase inhibitor 2A	P42771	GO:0005515	protein binding	0.68	-0.23
LPP	LIM domain containing preferred translocatio	Q93052	GO:0005829	cytosol	0.5	-0.93
DYNLT3	dynein light chain Tctex-type 3	P51808	GO:0005634	nucleus	0.43	-0.48
SCLY	selenocysteine lyase	Q96I15	GO:0016829	lyase activity	0.39	-0.41
DIAPH2	diaphanous related formin 2	O60879	GO:0031267	small GTPase binding	0.36	-0.53
CHD4	chromodomain helicase DNA binding protein	Q14839	GO:0003677	DNA binding	0.35	-0.15
TAOK3	TAO kinase 3	Q9H2K8	GO:0005524	ATP binding	0.3	-0.15
NOC3L	NOC3 like DNA replication regulator	Q8WTT2	GO:0005634	nucleus	0.25	-0.26
PRKAR2A	protein kinase cAMP-dependent type II regulat	Q8WTT2	GO:0005952	cAMP-dependent protein kinase complex	0.24	-0.60
CALM1	calmodulin 1	P0DP23	GO:0005509	calcium ion binding	0.2	-0.18
CXXC1	CXXC finger protein 1	Q9P0U4	GO:0003677	DNA binding	-0.21	0.14
PEX3	peroxisomal biogenesis factor 3	P56589	GO:0007031	peroxisome organization	-0.24	0.19
NDUFB5	NADH:ubiquinone oxidoreductase subunit B5	O43674	GO:0005739	mitochondrion	-0.25	0.30
PLEKHA8	pleckstrin homology domain containing A8	Q96JA3	GO:0005737	cytoplasm	-0.31	0.16
PPP3CA	protein phosphatase 3 catalytic subunit alpha	Q08209	GO:0033192	calmodulin-dependent protein phosphatase ac	-0.33	0.15
GOLM1	golgi membrane protein 1	Q8NBJ4	GO:0005794	Golgi apparatus	-0.43	0.16
JAM3	junctional adhesion molecule 3	Q9BX67	GO:0090138	regulation of actin cytoskeleton organization by	-0.43	0.15
MATR3	matrin 3	P43243	GO:0003676	nucleic acid binding	-0.48	0.14
ZFP36L2	ZFP36 ring finger protein like 2	P47974	GO:0046872	metal ion binding	-0.49	0.29
OSBPL1A	oxysterol binding protein like 1A	Q9BXW6	GO:0008289	lipid binding	-0.59	0.36
ZNF521	zinc finger protein 521	Q96K83	GO:0005634	nucleus	-0.69	0.93
ZMIZ1	zinc finger MIZ-type containing 1	Q9ULJ6	GO:0008270	zinc ion binding	-0.77	0.19
CDON	cell adhesion associated, oncogene regulated	Q4KMG0	GO:0005515	protein binding	-0.84	1.39
SH3BP5	SH3 domain binding protein 5	O60239	GO:0035556	intracellular signal transduction	-0.94	0.63
GPRC5B	G protein-coupled receptor class C group 5 me	Q9NZH0	GO:0007186	G protein-coupled receptor signaling pathway	-2.74	0.34
PDE1A	phosphodiesterase 1A	P54750	GO:0046069	cGMP catabolic process	-3.33	0.36
TMTC1	transmembrane O-mannosyltransferase target	Q8IUR5	GO:0005515	protein binding	-3.6	1.34

Supplementary Figure 1. Significant genes in Proband 1 and those identifed as reciprocal with knockout MIDEAS MEFs. a) Volcano plot of log2 fold change vs absolute Z score following RNA sequencing of Proband 1 compared with 30 control fibroblast lines. The number of significant genes (Fold change >1.2 and absolute zScore >2) that go down (green) and up (blue) are indicated. b) Table of reciprocal genes identified between Proband 1 and knockout MIDEAS MEFs. The log2 Fold change is shown.

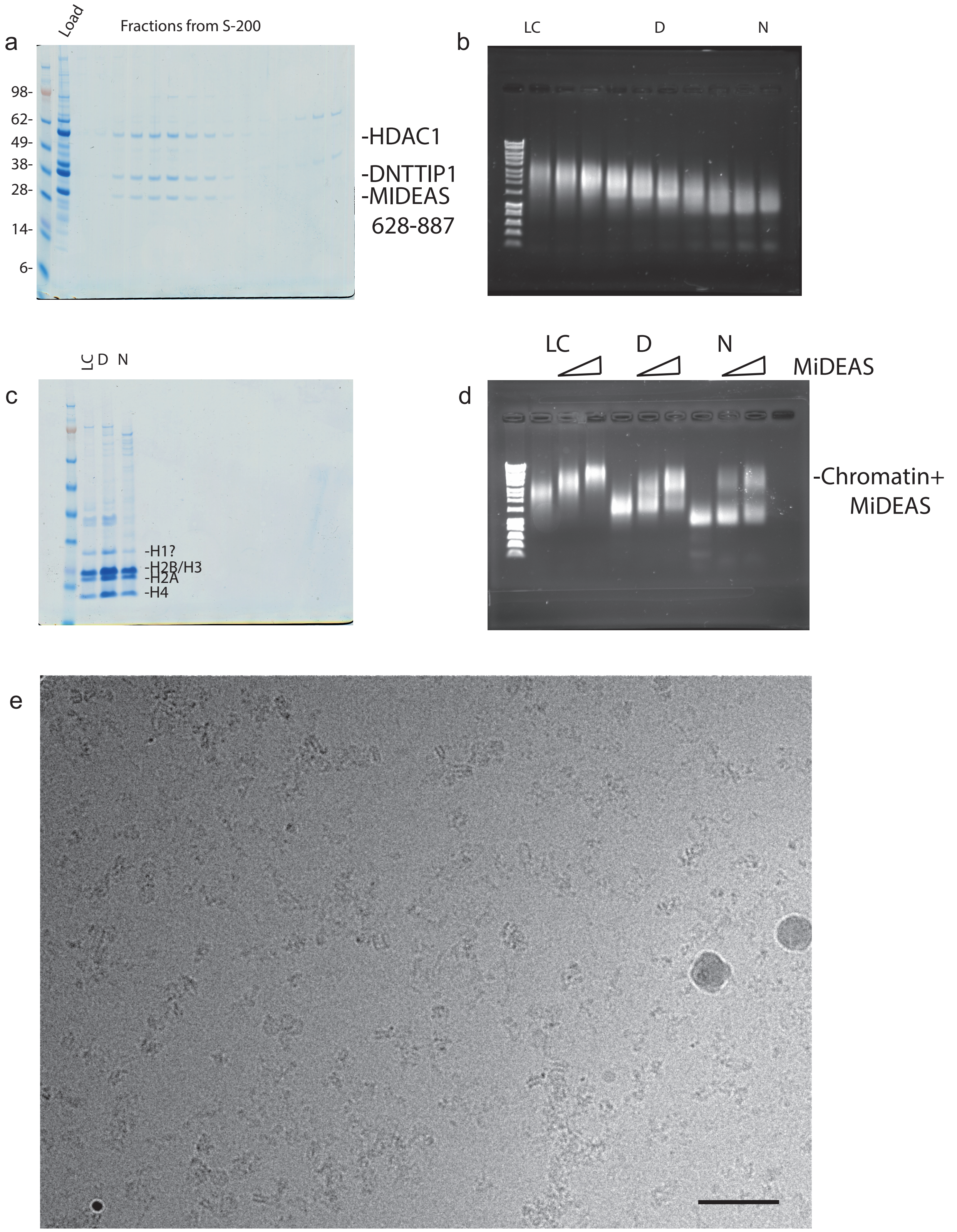

Supplementary Figure 2. Purification of chromatin from HEK293F cells and interaction with the MiDAC complex. a) SDS PAGE of fractions from Superdex S200 column of MiDAC complex expressed in HEK293F cells. "Load" is the concentrated complex before the column. b)Fractions of digested chromatin from HEK293F cells purified on a Superose 6 column on a 0.7% agarose 0.5xTB gel visualised with ethidium bromide. c) SDS PAGE of fractions of long chromatin (LC), dimer (D) and nucleosome (N). d) EMSA of the MiDAC complex binding to fractions of chromatin purified on the Superose 6 column. e) Micrograph from cryoSPARC, the scalebar is 50 nm.

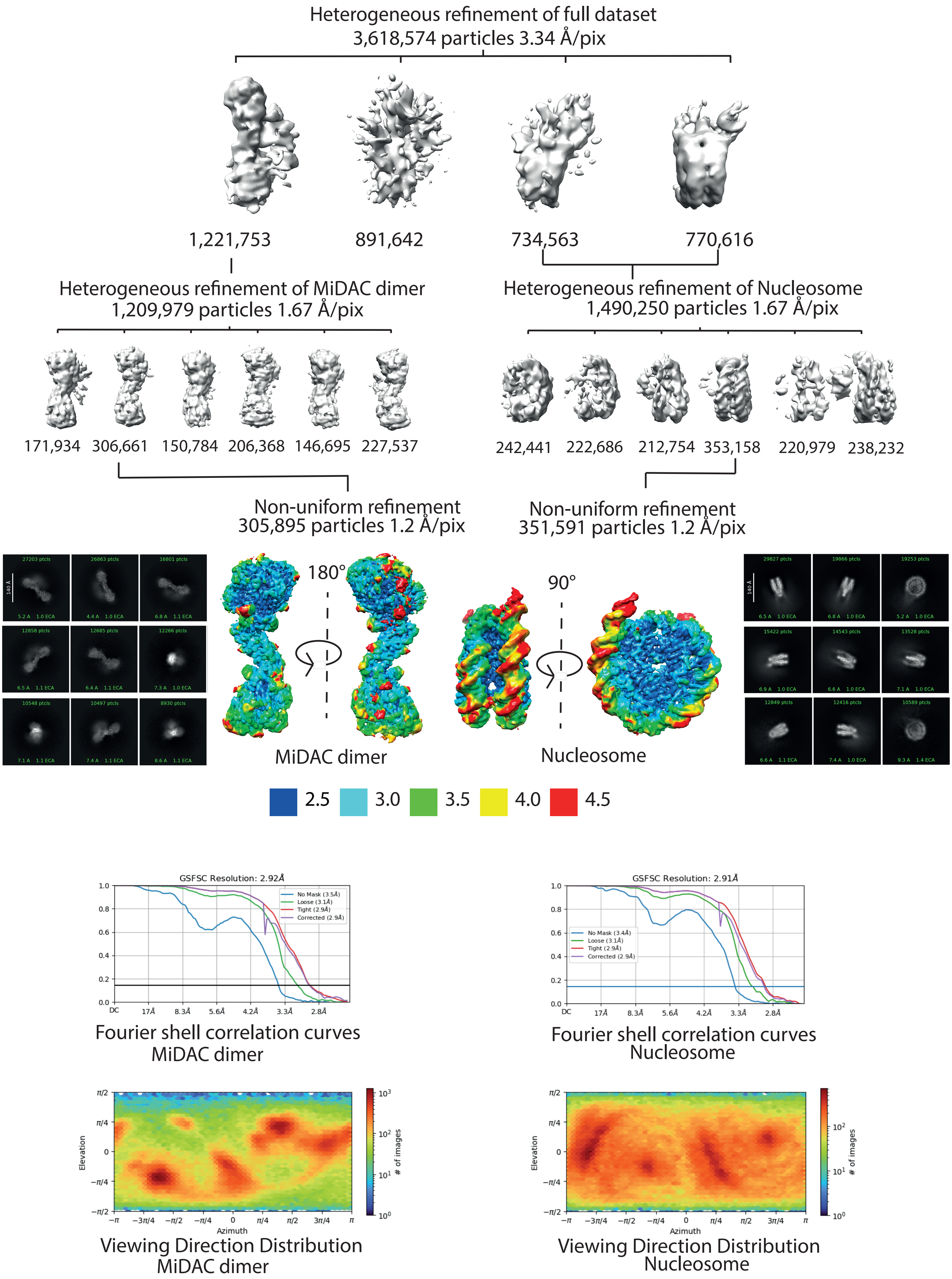

Supplementary Figure 3. Data processing in CryoSparc.

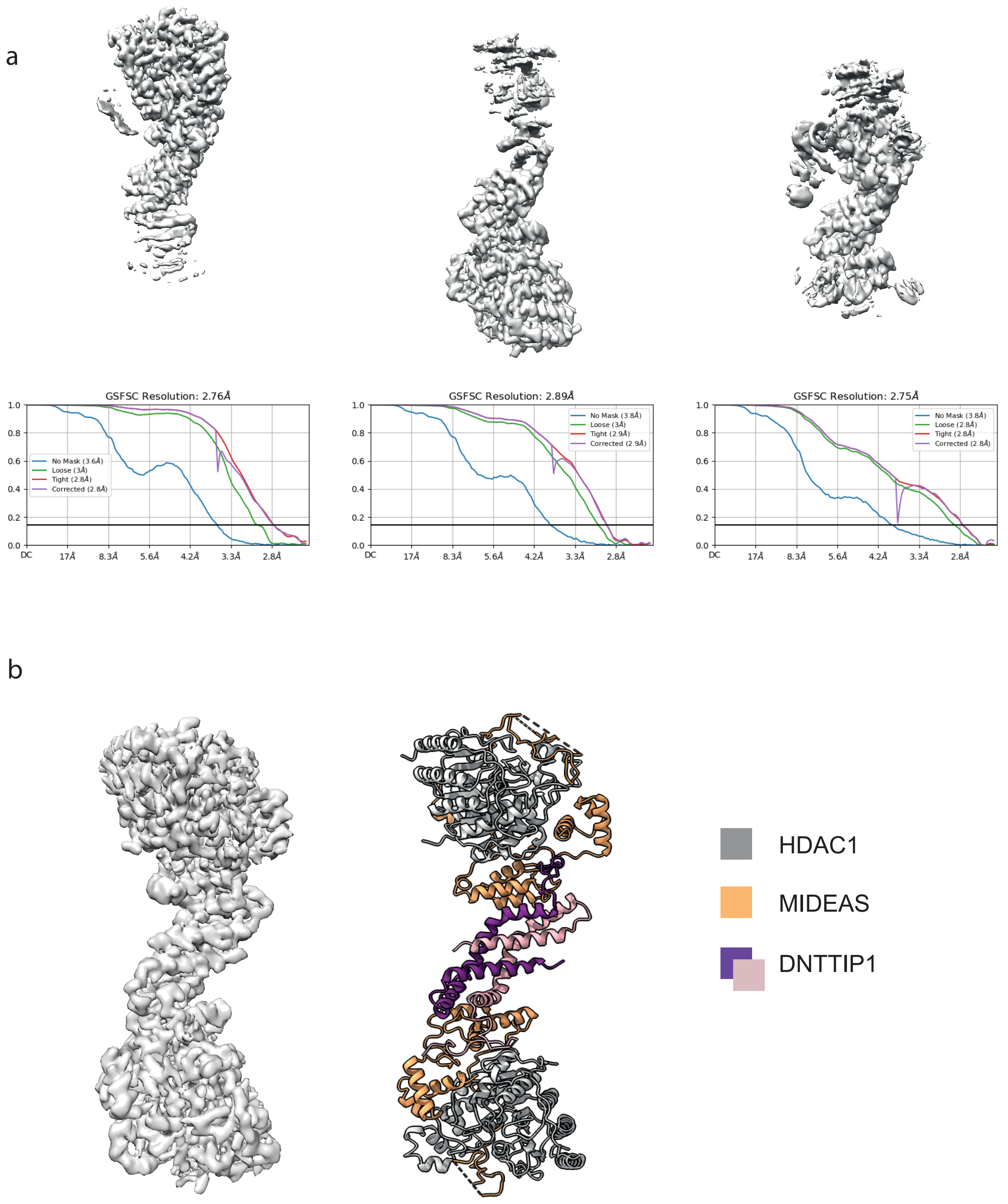

Supplementary Figure 4. Combining focussed maps. a) Individual focussed maps calculated in CryoSparc with the gold-standard Fourier shell correlation curves shown below. b) Combined focussed map calculated using Phenix. Map contour level is 10 in Chimera.

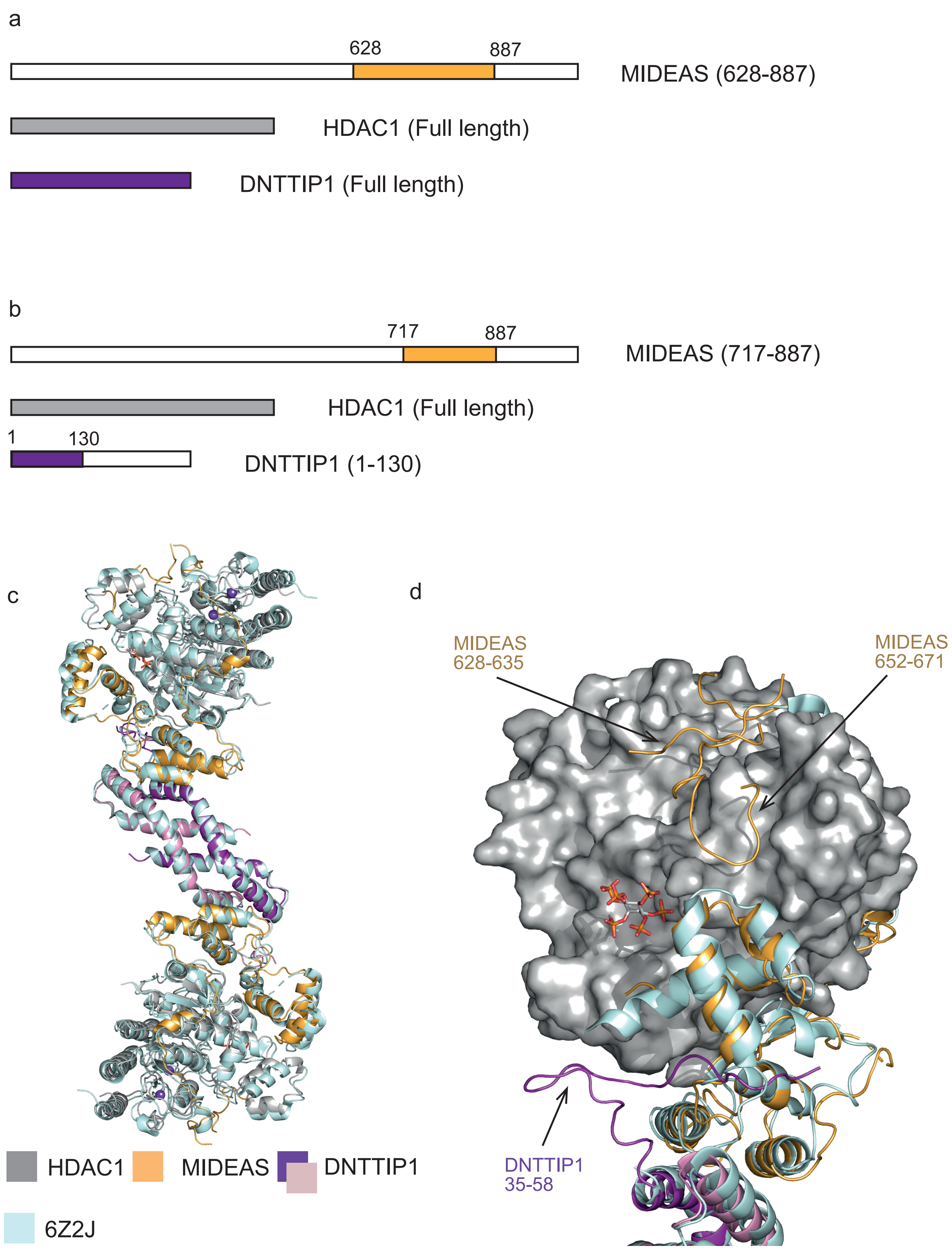

Supplementary Figure 5. Comparison of 2.9 Å structure with our previously published 4.0 Å structure 6Z2J. a) Constructs used in this study. b) Constructs used in previous study. c) Superimposition of both structures. d) Extra loops not seen in the previous structure are labelled.

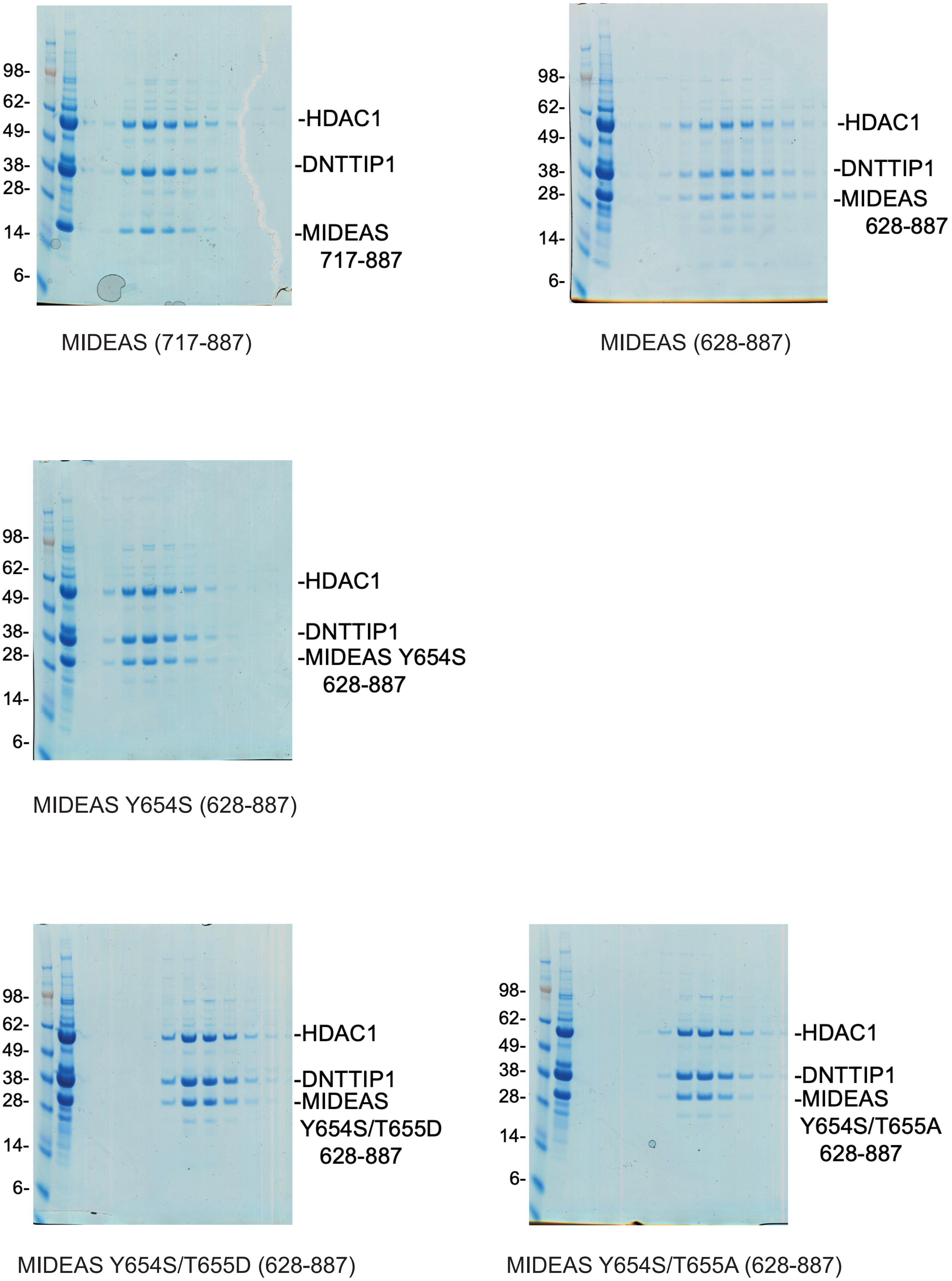

Supplementary Figure 6. SDS-PAGE gels of Superdex S-200 gel filtration purification of the complexes containing full length HDAC1, full length DNTTIP1 with various MIDEAS constructs.

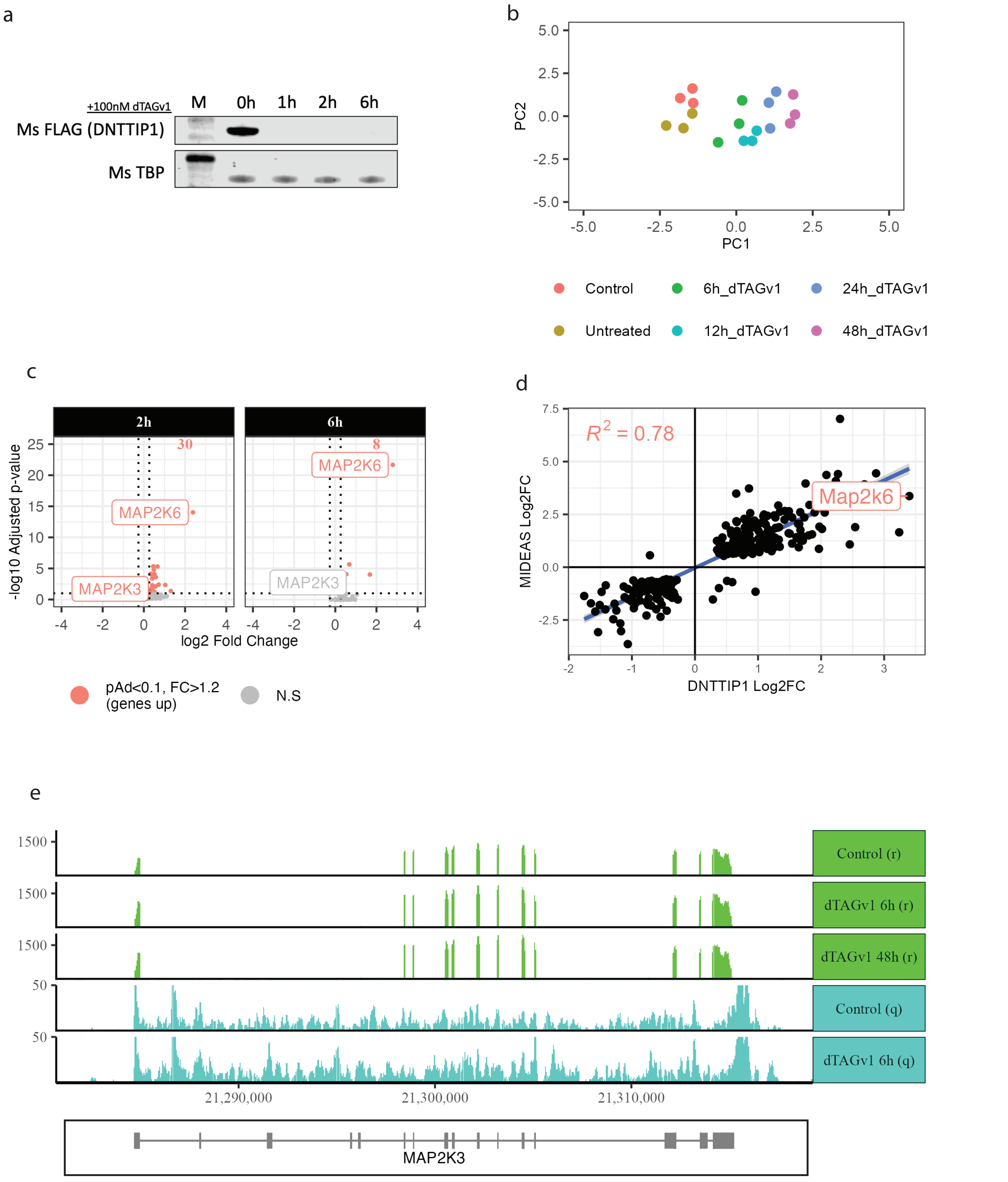

Supplementary Figure 7. Increases in MAP2K6 expression is reflected in increased transcription
a) Western blot showing time course of knockdown of DNTTIP1-FKBP/FLAG with dTAG^{v}-1 treatment. The blot was repeated three times with similar results b) PCA plot of the control and dTAG^{v}-1 treated replicates from HCT116 cells at various times. c) Volcano plot of fold change vs pAdj for qPRO sequencing. d) Shared differentially regulated genes from MIDEAS and DNTTIP1 KO MEF datasets showing MAP2K6. e) Gene locus of MAP2K3 showing counts from (r) RNAseq and (q) qPROseq experiments.

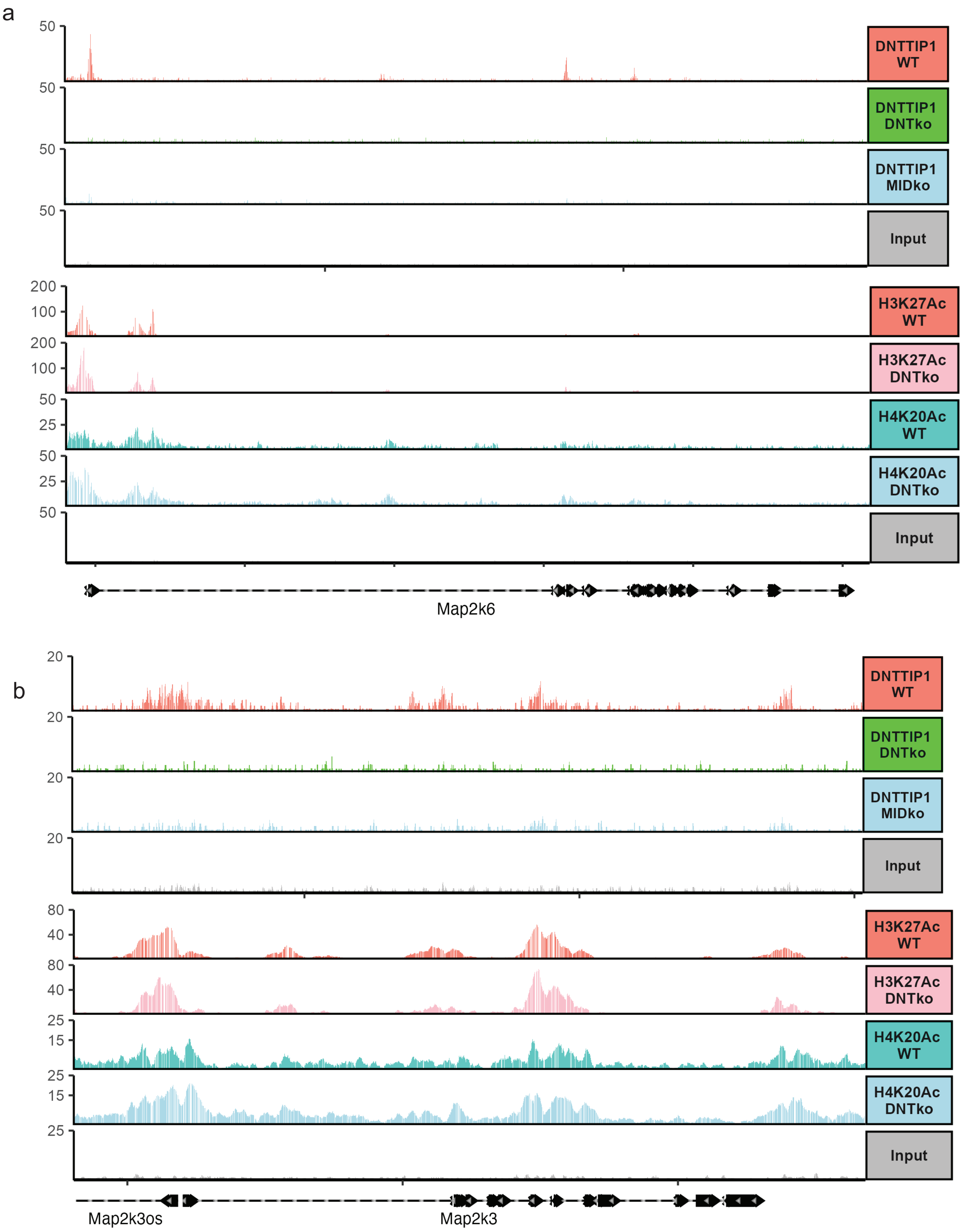

Supplementary Figure 8. Regulation of MAP2K6 and MAP2K3 by DNTTIP1. Pileup tracks of (a) MAP2K6 and (b) MAP2K3 ChIP-sequencing for DNTTIP1 from wildtype, DNTTIP1 knockout, and MIDEAS knockout mouse embryonic stem cells (mESCs), and for H3K27ac and H4K20ac from wildtype and DNTTIP1 knockout mESCs. DNTTIP1 ChIP data taken from Mondal et al., 2020 (PMID: 32297854; GSE131061) and acetylation ChIP data taken from Wang et al., 2022 (PMID: 35820704; GSE190323).

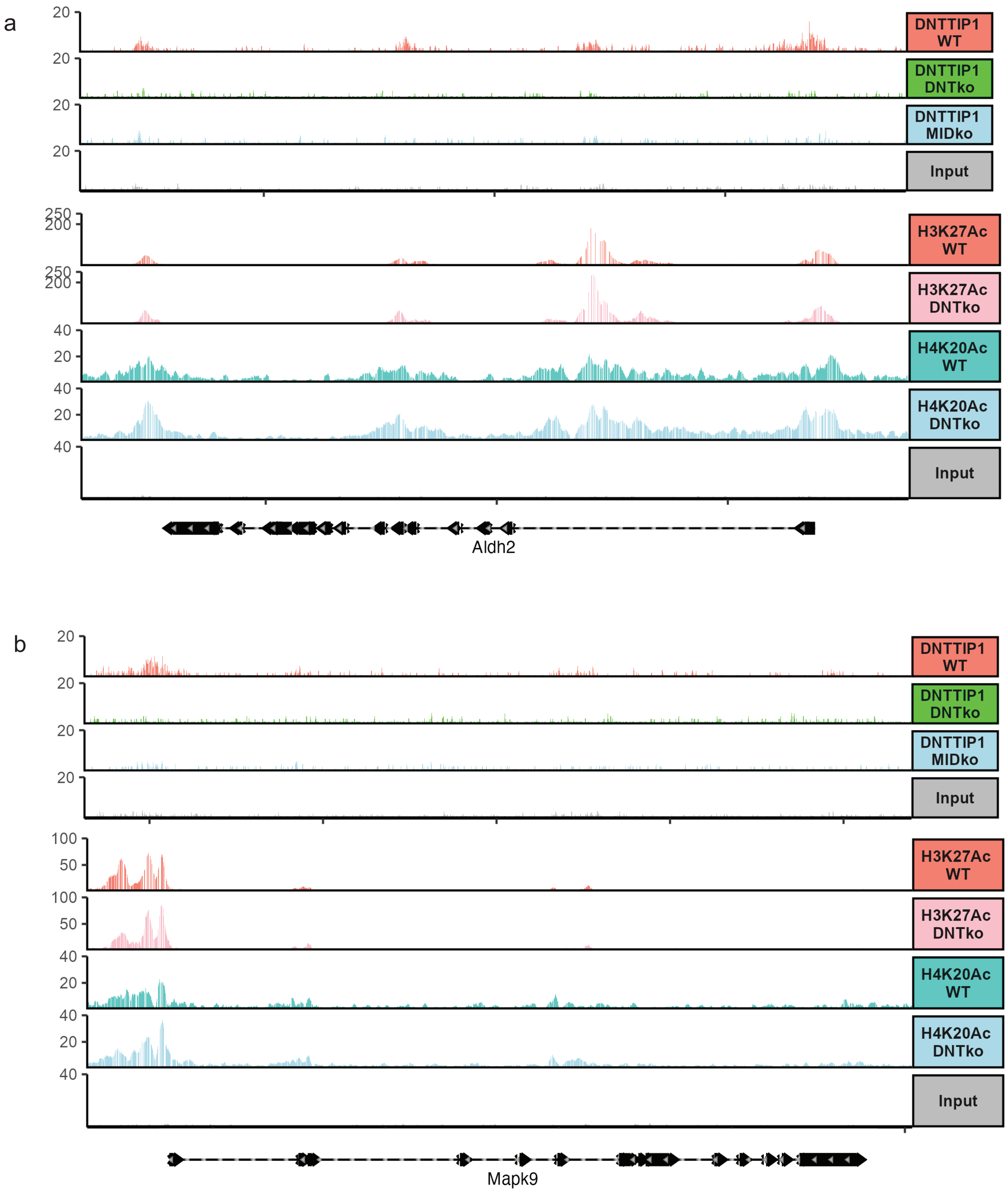

Supplementary Figure 9. Regulation of reciprocal genes by DNTTIP1. Pileup tracks of (a) ALDH2 and (b) MAPK9 representative of genes repressed in the Proband 1 and activated in the DNTTIP1 depleted datasets. ChIP-sequencing for DNTTIP1 from wildtype, DNTTIP1 knockout, and MIDEAS knockout mouse embryonic stem cells (mESCs), and for H3K27ac and H4K20ac from wildtype and DNTTIP1 knockout mESCs. DNTTIP1 ChIP data taken from Mondal et al., 2020 (PMID: 32297854; GSE131061) and acetylation ChIP data taken from Wang et al., 2022 (PMID: 35820704; GSE190323).

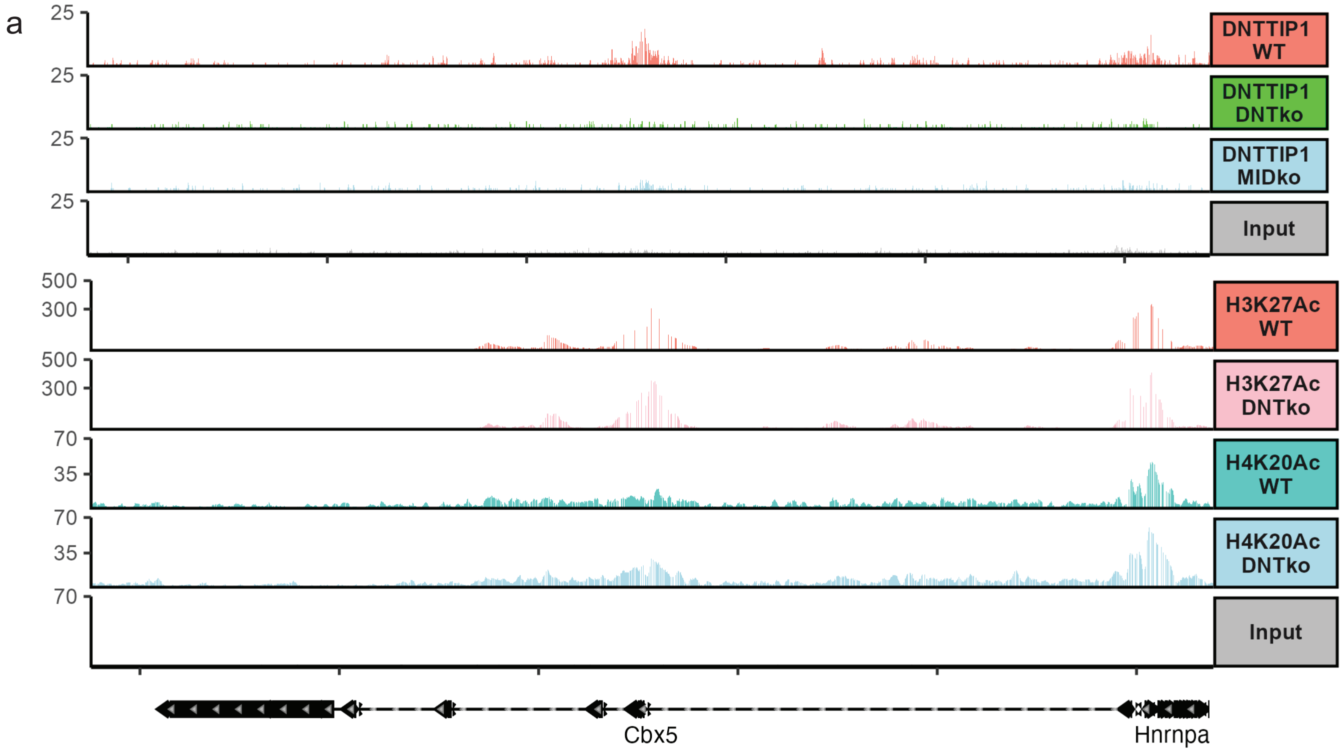

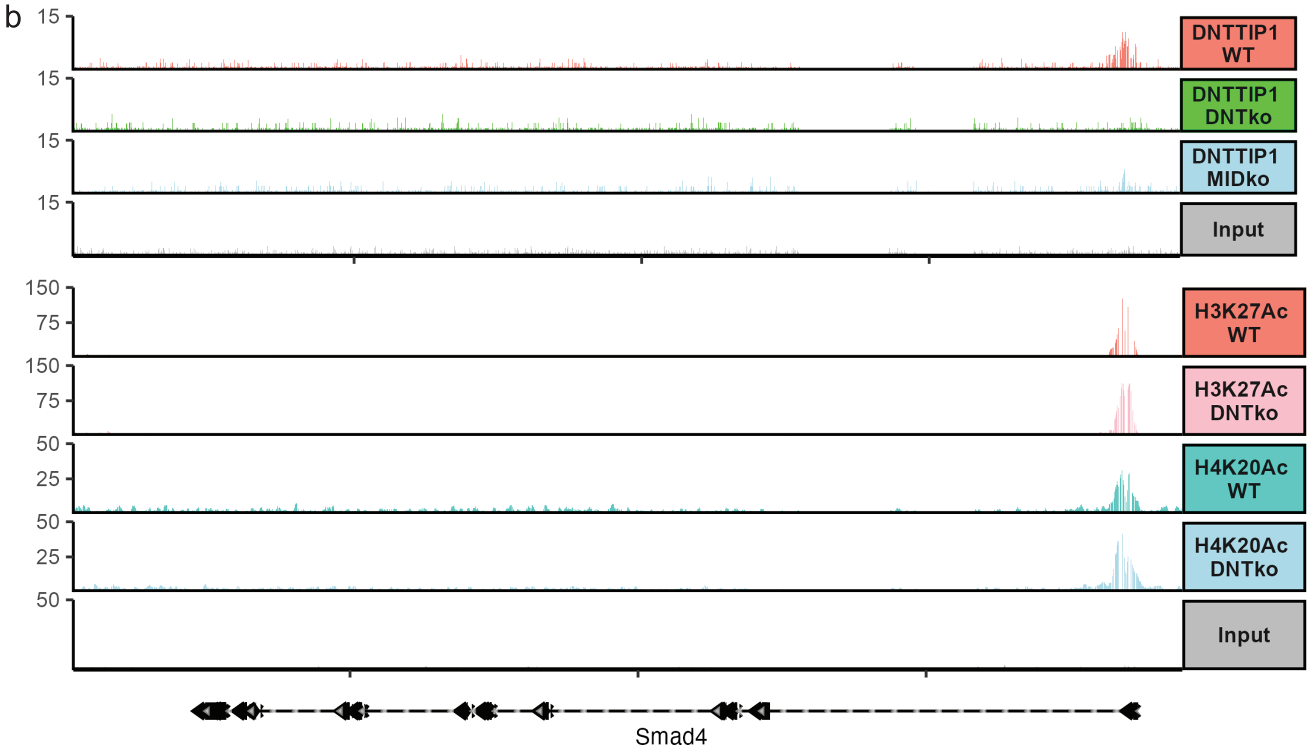

Supplementary Figure 10. Regulation of reciprocal genes by DNTTIP1. Pileup tracks of (a) CBX5 and (b) SMAD4 representative of genes activated in the Proband 1 and repressed in the DNTTIP1 depleted datasets. ChIP-sequencing for DNTTIP1 from wildtype, DNTTIP1 knockout, and MIDEAS knockout mouse embryonic stem cells (mESCs), and for H3K27ac and H4K20ac from wildtype and DNTTIP1 knockout mESCs. DNTTIP1 ChIP data taken from Mondal et al., 2020 (PMID: 32297854; GSE131061) and acetylation ChIP data taken from Wang et al., 2022 (PMID: 35820704; GSE190323).

HPO terms

Proband 1:
HP:0045025, Narrow palpebral fissure
HP:0000508, Ptosis
HP:0000426, Prominent nasal bridge
HP:0009765, Low hanging columella
HP:0000160, Narrow mouth
HP:0000347, Micrognathia
HP:0000220, Velopharyngeal insufficiency
HP:0000338, Hypomimic face
HP:0000407, Sensorineural hearing impairment
HP:0000405, Conductive hearing impairment
HP:0001072, Thickened skin
HP:0002580, Volvulus
HP:0004322, Short stature
HP:0008873, Disproportionate short-limb short stature
HP:0005781, Contractures of the large joints
HP:0002828, Multiple joint contractures
HP:0001156, Brachydactyly
HP:0001249, Intellectual disability
HP:0001263, Global developmental delay
HP:0002579, Gastrointestinal dysmotility
HP:0002014, Diarrhea

Proband 2
HP:0045025, Narrow palpebral fissure
HP:0000508, Ptosis
HP:0000426, Prominent nasal bridge
HP:0009765, Low hanging columella
HP:0000160, Narrow mouth
HP:0000220, Velopharyngeal insufficiency
HP:0000338, Hypomimic face
HP:0000405, Conductive hearing impairment
HP:0004322, Short stature
HP:0005781, Contractures of the large joints
HP:0002828, Multiple joint contractures
HP:0001156, Brachydactyly
HP:0001249, Intellectual disability
HP:0001263, Global developmental delay
HP:0002014, Diarrhea
HP:0001537, Umbilical hernia
HP:0000717, Autism

Cryo-EM data collection, refinement and validation statistics

	MiDAC Dimer (EMD-53563; EMD-53564, EMD-53565, EMD-53566; EMD-53567) (PDB 9R4I)
**Data collection and processing**	
Magnification	105,000
Voltage (kV)	300
Electron exposure (e–/Å^2)	47.4
Defocus range (μm)	-1.2 to -2.4
Pixel size (Å)	0.835
Symmetry imposed	P1
Initial particle images (no.)	3,618,574
Final particle images (no.)	305,895
Map resolution (Å)	2.9
FSC threshold	0.143
**Refinement**	
Initial model used (PDB code)	6Z2J
Model resolution (Å)	2.9
FSC threshold	0.143
Map sharpening *B* factor (Å^2)	109.2
Model composition	
Non-hydrogen atoms	10,475
Protein residues	1,284
Ligands	8
B factors (Å^2)	
Protein	100.7
Ligand	142.2
R.m.s. deviations	
Bond lengths (Å)	0.003
Bond angles (°)	0.536
Validation	
MolProbity score	1.65
Clashscore	6.48
Poor rotamers (%)	0.0
Ramachandran plot	
Favored (%)	95.72
Allowed (%)	4.12
Disallowed (%)	0.16

Supplementary Table 2
