# Supplementary material for: A de novo missense variant in MIDEAS results in increased deacetylase activity of the MiDAC HDAC complex causing a neurodevelopmental syndrome
Source: Nat Commun. 2025 Nov 25;16:10472. doi: 10.1038/s41467-025-65472-x (PMC12647621; doi:10.1038/s41467-025-65472-x)
Supplement: Supplementary file 2 — Description of Additional Supplementary Files [file 41467_2025_65472_MOESM2_ESM.pdf]

Description of Additional Supplementary Files

Supplementary Data 1: Table of Reciprocal Genes with annotation
