# Supplementary material for: A de novo missense variant in MIDEAS results in increased deacetylase activity of the MiDAC HDAC complex causing a neurodevelopmental syndrome
Source: Nat Commun. 2025 Nov 25;16:10472. doi: 10.1038/s41467-025-65472-x (PMC12647621; doi:10.1038/s41467-025-65472-x)
Supplement: Supplementary file 4 — Reporting Summary [file 41467_2025_65472_MOESM4_ESM.pdf]

nature portfolio

Corresponding author(s): Richard van Jaarsveld and John Schwabe

Last updated by author(s): 13.10.25

Reporting Summary

Nature Portfolio wishes to improve the reproducibility of the work that we publish. This form provides structure for consistency and transparency in reporting. For further information on Nature Portfolio policies, see our Editorial Policies and the Editorial Policy Checklist.

Statistics

For all statistical analyses, confirm that the following items are present in the figure legend, table legend, main text, or Methods section.

n/a	Confirmed	
☐	☒	The exact sample size (*n*) for each experimental group/condition, given as a discrete number and unit of measurement
☐	☒	A statement on whether measurements were taken from distinct samples or whether the same sample was measured repeatedly
☐	☒	The statistical test(s) used AND whether they are one- or two-sided<br>*Only common tests should be described solely by name; describe more complex techniques in the Methods section.*
☒	☐	A description of all covariates tested
☐	☒	A description of any assumptions or corrections, such as tests of normality and adjustment for multiple comparisons
☐	☒	A full description of the statistical parameters including central tendency (e.g. means) or other basic estimates (e.g. regression coefficient) AND variation (e.g. standard deviation) or associated estimates of uncertainty (e.g. confidence intervals)
☐	☒	For null hypothesis testing, the test statistic (e.g. *F*, *t*, *r*) with confidence intervals, effect sizes, degrees of freedom and *P* value noted<br>*Give P values as exact values whenever suitable.*
☒	☐	For Bayesian analysis, information on the choice of priors and Markov chain Monte Carlo settings
☒	☐	For hierarchical and complex designs, identification of the appropriate level for tests and full reporting of outcomes
☐	☒	Estimates of effect sizes (e.g. Cohen's *d*, Pearson's *r*), indicating how they were calculated

Our web collection on statistics for biologists contains articles on many of the points above.

Software and code

Policy information about availability of computer code

Data collection	Patient exome sequencing was acquired through clinical sequencing efforts.<br><br>Patient fibroblasts RNA seq: RNA seq on PolyA enriched RNA was performed on a Novaseq X plus (Illmina), 150 bp paired-end for index patient and all controls.<br><br>RNA sequencing (MIDEAS model systems): Sequenced by Novogene using NovaSeq6000/NovaSeq Xplus with 150bp paired end sequencing to a depth of 20 million reads.<br><br>Nascent sequencing: performed by Novogene using a NovaSeq 6000 S4 using 150 base-pair pair-end sequencing to a minimum depth of 50 million reads.<br><br>CryoEM data collection: EPU 3.4
Data analysis	Patient exome sequencing data was analyzed using standardized diagnostic pipelines.<br><br>Patient RNA sequencing: Quality Check: FASTQC(v0.11.8), PICARD (v2.22.0); Alignement:STARalign(v2.7.3) to GRCh38,gencode (v43);count files:featureCounts (v2.0.1).Analysis: OUTRIDER (v1.18.1)

RNA sequencing (MIDEAS model systems):
Processing:
Quality check: FASTQC (v0.12.1); Alignment: HISAT2 (v12.3.0) to GRCh38 release 111; file formating: samtools (v1.17); count files: LiBiNorm (v2.5; options: -z-r pos -l gene_name -s reverse) with GRCh38 release 111 gene transfer file.
Analysis: Performed using R (v4.41)
Calculation of significant genes: DESeq2 (v1.44.0) (Wald test with local fit type and BH method for correction). APEGLM (v1.26.1) was used to calculate shrinkage estimates
Software used to further analyze and visualize data: ggplot2 (v3.5.1), ggh4x (v0.2.8), ggcorrplot (v0.1.4.1), ComplexHeatmap (v2.20.0), ggcoverage (v1.4.0), TopGO (v2.56.0).

Nascent sequencing:
Quality check: fastqc (v0.12.1); Adaptor removal: Fastp (v0.23.2; options: --umi --stdout --umi_loc=per_read --umi_len=6 -c --overlap_len_require 15). rRNA removal: bowtie2 (v12.3.0; options: --fast-local --un-conc -- interleaved); alignment: Bowtie2 (options: --local --sensitive-local) to hg38; file formatting: samtools (v1.17); deduplication: UMI-tools (v1.0.1; options: --umi-separator=":" --paired); bigwig file generation: Deeptools (v3.5.1) bamCoverage (options: --skipNonCoveredRegions --binSize 1 --normalizeUsing None --Offset 1 --samFlagInclude 82 (forward) or 98 (reverse))
Analysis: Performed using R (v4.41)
Calculation of significant genes across gene bodies: DESeq2 (v1.44.0) (Wald test with local fit type and BH method for correction). APEGLM (v1.26.1) was used to calculate shrinkage estimates
Software used to further analyze and visualize data: ggplot2 (v3.5.1), ggh4x (v0.2.8), ggcoverage (v1.4.0), ggVennDiagram (v1.5.2).

CryoEM data analysis: CryoSparc v4.4.1, Phenix 1.21.1, Modelangelo 0.0.1, Coot 0.9.6
Protein mass spectrometry: PEAKS Studio X (BSi, Canada) and Scaffold (Proteome Software, Oregon)
NMR data analysis: Topspin version 3.6.5, Prism 10.4.0 (GraphPad)

For manuscripts utilizing custom algorithms or software that are central to the research but not yet described in published literature, software must be made available to editors and reviewers. We strongly encourage code deposition in a community repository (e.g. GitHub). See the Nature Portfolio guidelines for submitting code & software for further information.

Data

Policy information about availability of data

All manuscripts must include a data availability statement. This statement should provide the following information, where applicable:

- Accession codes, unique identifiers, or web links for publicly available datasets
- A description of any restrictions on data availability
- For clinical datasets or third party data, please ensure that the statement adheres to our policy

Patient exome sequencing and fibroblast transcriptomic data are not publicly available due to privacy/ethical restrictions.

Raw and processed RNA sequencing and nascent sequencing files have been submitted to GEO (Project Accession: GSE297959; GSE297958).

The coordinates for the MiDAC model are available from the PDB under the accession code 9R4I. The EM maps are available from EMDB under the following accession codes: consensus map EMD-53563; focussed maps EMD-53564, EMD-53565, EMD-53566; combined focussed map EMD-53567.

The mass spectrometry proteomics data have been deposited to the ProteomeXchange Consortium via the PRIDE partner repository with the dataset identifier PXD067661 and 10.6019/PXD067661

Research involving human participants, their data, or biological material

Policy information about studies with human participants or human data. See also policy information about sex, gender (identity/presentation), and sexual orientation and race, ethnicity and racism.

Reporting on sex and gender	In this study two participants were included based on the presence of a specific genetic variant and similar phenotype. Sex and gender of the participants was not specifically considered in the study design.<br>Informed consent was collected to share individual-level data of the participants.<br>Due to the number of participants no sex- or genderbased analyses were performed.
Reporting on race, ethnicity, or other socially relevant groupings	In this study two participants were included based on the presence of a specific genetic variant and similar phenotype. Ethnicity or socioeconomic status was not taken into consideration. Due to the number of participants no ethnicity based analyses were performed.
Population characteristics	Individual data on age, genotype and phenotype is provided for the participants. Informed consent was collected to share individual-level data of the participants.

Recruitment	The patients with a variant in the MIDEAS-gene were informed about the study and they and their parents were asked for Informed Consent by their treating physicians.
Ethics oversight	This study does not fall under the scope of the Dutch Medical Research Involving Human Subjects Act (WMO). It therefore does not require approval from an accredited medical ethics committee in the Netherlands.<br>However, in the UMC Utrecht, an independent quality check has been carried out to ensure compliance with legislation and regulations (regarding Informed Consent procedure, data management, privacy aspects and legal aspects), see enclosed forms.

Note that full information on the approval of the study protocol must also be provided in the manuscript.

Field-specific reporting

Please select the one below that is the best fit for your research. If you are not sure, read the appropriate sections before making your selection.

[x] Life sciences [ ] Behavioural & social sciences [ ] Ecological, evolutionary & environmental sciences

For a reference copy of the document with all sections, see nature.com/documents/nr-reporting-summary-flat.pdf

Life sciences study design

All studies must disclose on these points even when the disclosure is negative.

Sample size	No sample size calculation was performed. All individuals with a specific MIDEAS variant were included (total=2)
Data exclusions	No individuals were excluded from the study.
Replication	Not applicable
Randomization	Participants were included based on the presence of a specific genetic variant and similar phenotype. Therefore randomization was nog feasible in this study.
Blinding	Participants were included based on the presence of a specific genetic variant and similar phenotype. Therefore blinding was not feasible in this study.

Reporting for specific materials, systems and methods

We require information from authors about some types of materials, experimental systems and methods used in many studies. Here, indicate whether each material, system or method listed is relevant to your study. If you are not sure if a list item applies to your research, read the appropriate section before selecting a response.

Materials & experimental systems

n/a	Involved in the study	
[ ]	[x]	Antibodies
[ ]	[x]	Eukaryotic cell lines
[x]	[ ]	Palaeontology and archaeology
[x]	[ ]	Animals and other organisms
[ ]	[x]	Clinical data
[x]	[ ]	Dual use research of concern
[x]	[ ]	Plants

Methods

n/a	Involved in the study	
[x]	[ ]	ChIP-seq
[x]	[ ]	Flow cytometry
[x]	[ ]	MRI-based neuroimaging

Antibodies

Antibodies used	MAP2K6 (MEK6) - Host species: Mouse; target species: Human; clone: 3H12C8; Source: Invitrogen #MA5-15808; lot:ZB4215171<br>phospho MAP2K6 (pMEK6) - Host species: Rabbit; target species: Human. Source: Invitrogen #PA5-37702; lot: ZB44216621<br>FLAG - Host species: Mouse; target: 3x FLAG; clone: M2. Source: Sigma #F1804; lot: 1003310514<br>TBP - Host species: Mouse; target species: Human; clone: 58C9 . Source: SantaCruz #SC-421; lot: KO422<br>alpha Tubulin - Host Species: Rabbit; target species: Human. Source: Abcam #AB4074; lot: GR3416761<br>IRDye680RD Goat anti-mouse IgG secondary antibody. Source: LicorBio #926-68070; lot: D30418-05<br>IRDye800CW Goat anti-rabbit IgG secondary antibody. Source: LicorBio #926-32211; lot: D40625-05
Validation	All antibodies are supplier validated or highly cited in other publications. The FLAG antibody was additionally shown to be specific based on knockdown of protein.

Eukaryotic cell lines

Policy information about cell lines and Sex and Gender in Research

Cell line source(s)	Patient Fibroblasts - Fibroblasts were cultured from skin biopsies for index and all control samples. All fibroblasts have been obtained for diagnostic purposes.<br><br>HCT 116 colon carcinoma cell line isolated from a male - Sigma Aldrich #91091005-1VL<br><br>HEK293F cells for protein expression for CryoEM - Thermo Fisher
Authentication	No authentication was carried out in house for the HCT 116 cell line or HEK293F. However, authentication has been performed by the supplier.
Mycoplasma contamination	HCT 116 cells were regularly tested for Mycoplasma contamination using PCR with primers against 16s ribosomal RNA. All human fibroblasts were tested negative before sequencing.
Commonly misidentified lines (See ICLAC register)	N/A

Clinical data

Policy information about clinical studies

All manuscripts should comply with the ICMJE guidelines for publication of clinical research and a completed CONSORT checklist must be included with all submissions.

Clinical trial registration	Not applicable
Study protocol	*Note where the full trial protocol can be accessed OR if not available, explain why.*
Data collection	*Describe the settings and locales of data collection, noting the time periods of recruitment and data collection.*
Outcomes	*Describe how you pre-defined primary and secondary outcome measures and how you assessed these measures.*

Plants

Seed stocks	Not applicable
Novel plant genotypes	*Describe the methods by which all novel plant genotypes were produced. This includes those generated by transgenic approaches, gene editing, chemical/radiation-based mutagenesis and hybridization. For transgenic lines, describe the transformation method, the number of independent lines analyzed and the generation upon which experiments were performed. For gene-edited lines, describe the editor used, the endogenous sequence targeted for editing, the targeting guide RNA sequence (if applicable) and how the editor was applied.*
Authentication	*Describe any authentication procedures for each seed stock used or novel genotype generated. Describe any experiments used to assess the effect of a mutation and, where applicable, how potential secondary effects (e.g. second site T-DNA insertions, mosiacism, off-target gene editing) were examined.*
